# C1q deletion exacerbates stress-induced learned helplessness behavior and induces neuroinflammation in mice

**DOI:** 10.1038/s41398-022-01794-4

**Published:** 2022-02-01

**Authors:** Amit Kumar Madeshiya, Carl Whitehead, Ashutosh Tripathi, Anilkumar Pillai

**Affiliations:** 1 Pathophysiology of Neuropsychiatric Disorders Program, Faillace Department of Psychiatry and Behavioral Sciences, McGovern Medical School, The University of Texas Health Science Center at Houston (UTHealth), Houston, TX USA; 2 Department of Psychiatry and Health Behavior, Medical College of Georgia, Augusta University, Augusta, GA USA; 3 Research and Development, Charlie Norwood VA Medical Center, Augusta, GA USA

**Keywords:** Molecular neuroscience, Depression

## Abstract

Increased levels of pro-inflammatory cytokines have been reported in postmortem brain samples and in the blood of depressed subjects. However, the inflammatory pathways that lead to depressive-like symptoms are not well understood. Using the learned helplessness (LH) model of depression, we examined the role of C1q, the initiator of classical complement pathway in mediating stress-induced depressive-like behavior in mice. We observed no significant changes in social behavior, despair behavior, spatial memory, and aggressive behavior between the wild type (WT) and C1q knockout (KO) mice. However, C1q deletion exacerbated the inescapable electric foot shock-induced learned helplessness behavior in mice. We found significant reductions in C1q mRNA levels in the prefrontal cortex (PFC) of WT helpless mice as compared to the naïve mice. Increased levels of pro-inflammatory cytokines were found in the PFC of C1q KO mice. These findings suggest that classical complement pathway-mediated learned helplessness behavior is accompanied by neuroinflammatory changes under stressful conditions.

## Introduction

Depression is a leading cause of disability and is a significant contributor to the increasing rate of suicide [1, 2]. Both genetic and environmental factors contribute to the pathophysiology of depression [3, 4]. Recent studies have found higher rate of depressive symptoms in many conditions known to have an inflammatory basis such as rheumatoid arthritis, asthma, and cardiovascular disease [5–7]. Similarly, increased levels of circulating inflammatory markers including cytokines and leukocytes are consistently found in depressed subjects [6, 8–13]. Studies have also reported increased neuro-immune and neuro-endocrine responses following chronic stress conditions which could result in the development of behavioral vulnerability and resilience depending upon the independent stage of these responses [14–17]. These findings suggest a significant role of immune system in the pathophysiology of depression.

There is an emerging interest in the field to investigate the relation between depression and the complement system. The complement system is composed of more than 30 proteins that collectively participate in the host defense mechanism by regulating inflammation [18]. The complement system is activated by three pathways, classical, lectin, and alternative pathway [19]. The classical pathway is activated by the C1 protein complex, composed of C1q, C1r, and C1s. The activated C1 complex promotes the cleavage of C3 to C3a and C3b. C3 is considered as the hub of the complement activation pathways to coordinate downstream immune responses [20]. Increased levels of components C3a and C5a have been found in patients suffering from bipolar disorder (BD) [21]. Also, higher levels of cerebrospinal fluid C5 levels were reported in the patients with major depression (MDD) compared with the healthy controls [22]. Similarly, our earlier study found an increase in C3 mRNA levels in the prefrontal cortex (PFC) of depressed suicide subjects [23]. In addition, we found that inhibition of C3a signaling attenuates chronic stress-induced depressive-like behavior in mice [23–25].

Learned helplessness (LH) is a rodent model of depression with excellent face, construct, and predictive validity [26]. In LH, the organism has no control over the prior aversive events such as inescapable electric shocks [27, 28]. The LH is considered as one of the processes involved in the development of depression [27]. Further, studies have found similarities in the course of helplessness and its response to antidepressants between the rodent LH model and clinical depression [29].

In the present study, we investigated the role of classical complement pathway in LH behavior. C1q is the initiator of the classical pathway and an increase of C1q in activated microglia has been shown to promote the secretion of pro-inflammatory cytokines such as interleukin-6 (IL-6) and tumor necrosis factor-alpha (TNFα) [30]. We examined whether changes in C1q and LH are associated with alterations in inflammatory markers by examining classically activated (M1) and alternatively activated (M2) states of macrophages/microglia.

## Material and methods

### Animals

Adult (8 weeks old) male C1qa^−/−^(C1q KO; strain #031675) and their age-matched wild type (WT) (C57BL/6J) mice were purchased from The Jackson Laboratory. All the animals were housed and maintained in the animal housing facility at Charlie Norwood VA Medical Center, Augusta, GA or Augusta University, Augusta, GA. Mice were housed in groups of 5 in standard polypropylene cages in 12 h light-dark cycle in compliance with the US National Institute of Health guidelines, and approved by Augusta University and Charlie Norwood VA Medical Center animal welfare guidelines. Mice were assigned to experimental groups based on their genotype, and treatment studies were performed in a blinded manner.

### Behavior tests

All behavior tests were performed in a separate behavior room facilitated with ambient lighting, constant background sound, temperature, and pressure. For the suitability of behavior room, the aforesaid environmental conditions of the room were always monitored prior to the transfer of animals from animal facility, as well as kept constant during the experiment. Animals were allowed to habituate in the behavioral room for at least 1 h before testing. All behavioral experiment recordings were scored blind to treatment. We used 7–10 mice per group for behavior tests.

### Three-chamber test

A three-chamber apparatus made of clear Plexiglas was used. Each dividing partition of the chambers has openings on each wall for free access to the other two chambers. The dimension of each chamber was 19 cm × 45 cm × 22 cm. Two identical wire containers (9 cm diameter and capable to house a single mouse) were placed vertically in the side chambers of the apparatus. The wire containers were made in such a way to facilitate only air exchange but no physical contact between stranger mouse and test mouse. The test mouse was placed in the center chamber and allowed to move freely across all the chambers for 5 m to get acclimatize to the chambers. After 5 min acclimatization period, the age, sex, and background-matched stranger mouse was placed in one of the wire containers. The test mouse was allowed to freely move outside of the container for an additional 5 m. Time spent by the test mouse in chambers (stranger mouse chamber, empty chamber, and center chamber) was video recorded.

### Social interaction test

In this test, the stranger mouse was allowed to move freely across the entire three-chamber apparatus along with the test mouse so that the test mouse can interact physically. The interaction between the mice was defined as close physical contact, nose-to-nose sniffing, ano-genital sniffing, and grooming. Time of interaction (initiated by the test mouse only) was video recorded for 5 m.

### Y-maze test

A Y-maze apparatus (Maze Engineers) was used to score the continuous and spontaneous alternation in mice. The Y-maze consists of three equal arms of 6 cm width and 32 cm length, and joined at 120°. Distal visual cues were placed in front of each arm of the Y-maze. A mouse was placed in the Y-maze and allowed to explore for 7 min. Mouse behavior was recorded using a webcam (C920, Logitech, Newark, CA) and analyzed by the ANY-Maze software (Stoelting, Wood Dale, IL). An entry occurs when the whole body (except for the tail) enters the arm, and an exit occurs if the whole body (except for the tail) exits from the arm. An alternating triad is considered when an animal consecutively entered three different arms. Because the maximum number of triads is the total number of arm entries minus 2, the score of alternation was calculated as [the number of alternating triads/(the total number of arm entries − 2)].

### Tail suspension test

The tail suspension test measures behavioral despair. Briefly, each mouse was suspended about 70 cm away from the floor. Suspension of each mouse was performed from 0.75 cm away from the tip of their tail, using adhesive tape. The test was recorded for 6 min with the help of a camera and the immobility time was scored manually.

### Resident intruder test

Aggressive behavior was examined by the resident intruder test. In this test, a stranger intruder mouse was placed into the home cage of the experimental mouse. The test was performed during the dark cycle. The intruder mouse was of the same background but relatively (~2 week) younger than the resident mouse. The behavior was recorded for 5 min and scored for various aggressive behaviors.

### Learned helplessness paradigm

Mice (*n* = 11 per group) received 100 inescapable electric foot shocks (EFSs) delivered at an intensity of 0.3 mA for a duration of 10 s and an unpredictable inter-shock interval of 5–99 s in shock chambers (chamber dimensions, 17 cm × 17 cm × 20 cm; Maze Engineers, USA) for 3 days. Control mice were placed in the shock chamber for the same amount of time with no inescapable shock exposure. Using the shuttle-box active avoidance task, LH was assessed at 24 h after the third training session. In this paradigm, mouse was placed on one side of a shuttle box and allowed to explore the shuttle box for 5 min. After the acclimatization period, mice were exposed to 30 trials of unpredictable but escapable foot shock delivered at an intensity of 0.3 mA for a maximum duration of 24 s and an unpredictable inter-shock interval of 30–60 s. The foot shock exposure stops when the mouse completely crosses to the other side of the box. A failure to cross to the other side to terminate the shock results in an escape failure. Since mice exposed to LH-inducing stimuli can exhibit stress-resilience without helplessness, both learned and non-learned helplessness (NLH) were calculated in this study. LH group consisted of mice with escape failures longer than the upper limit of the 95% confidence interval whereas those with failures shorter than the upper limit of the 95% confidence interval were considered as NLH.

### mRNA expression analysis (q-RT-PCR)

Total RNA from the PFC (*n* = 3–7) samples was isolated using a commercially available kit (SV RNA Isolation, Promega, Madison, WI, USA). All RNA samples were quantified using a Nanodrop. After the quantification, cDNA was prepared using iScript™ cDNA Synthesis Kit (Bio-Rad CA, USA). q-RT-PCR was performed on a Master Cycler (Quant Studio-7 Real-Time PCR Systems, Thermo Fisher Scientific, USA) using iTaqTM Universal SYBR^®^ Green Super mix (Bio-Rad, CA, USA). Primers were synthesized by Integrated DNA Technologies. Ct values of genes of interest were normalized to that of housekeeping gene, beta2-microglobulin (B2M). The list of primers used is given in Table S1.

### Cytokine array

Simultaneous quantification of cytokines in mouse sera (*n* = 5–6) and PFC (*n* = 5–7) samples was performed using LEGENDplex Mouse Inflammation Panel (13-plex) with V-bottom Plate (BioLegend Cat# 740446) according to the manufacturer’s instructions. Serum samples were diluted two-fold with assay buffer, and standards were mixed with Matrix solution (Biolegend) to account for additional components in the serum samples. Standards and samples were plated with capture beads for TNFα, Interferon-gamma (IFN-γ), IL-1α, IL-1β, IL-6, IL-10, IL-17A, IL-12p70, granulocyte-macrophage colony-stimulating factor (GM-CSF), IL-23, IFN-β, monocyte chemoattractant protein-1 (MCP-1), IL-27 and incubated for 2 h at room temperature on a plate shaker (800 rpm). After washing with wash buffer, the plate was incubated with detection antibodies on a shaker for 1 hour at room temperature. Following the incubation with antibodies, streptavidin R-phycoerythrin (SA-PE) was added and incubated for 30 min. Samples were acquired on CytoFLEX flow cytometer (Beckman Coulter Life Sciences). Standard curves and protein concentration were calculated using R package DrLumi [31] installed on R 3.5.2 (https://www.r-project.org/). The limit of detection was calculated as the average of background samples plus 2.5 × SD. Assay and data calculations were performed at the Immune Monitoring Shared Resource (Augusta University).

### Statistical analysis

No statistical methods were used to predetermine sample sizes in the current study, but our sample sizes are similar to those reported in previous publications [24, 25]. Data were presented as mean ± SEM. Data were analyzed using two-tailed Student’s t-test (for two-group comparisons) or Analysis of Variance (ANOVA; for multiple-group comparisons). Post hoc analyses were carried out using Tukey’s test. Grubbs’ test was performed to identify the significant outliers. All the statistical analyses and graph preparation were performed using GraphPad Prism 9.0.0. *p* < 0.05 was considered significant.

## Results

### C1q KO mice display no abnormalities in social interaction, despair behavior, spatial memory, and aggressive behavior

**Fig. 1:**
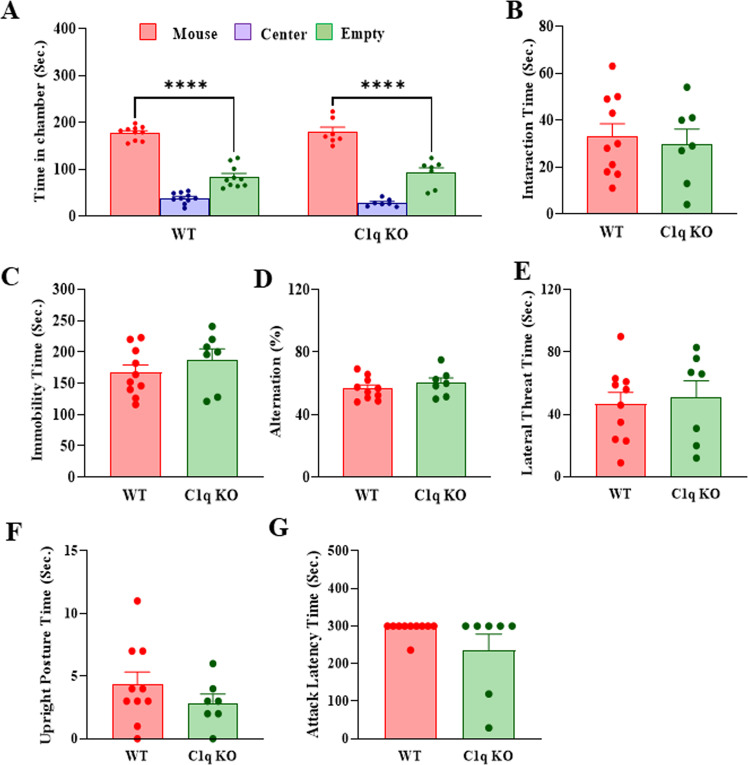
C1q KO mice display no abnormalities in social interaction, despair behavior, spatial memory and aggressive behavior. **A** Time in chamber in the three-chamber social interaction test. Two-way ANOVA, genotype X chamber interaction (F (2, 45) = 0.9543, *p* = 0.3927); ****p < 0.0001 (mouse chamber vs. empty chamber). **B** Reciprocal social interaction test. Unpaired t-test, *p* = 0.7044 vs WT. **C** Immobility time in tail suspension test. Unpaired t-test, *p* = 0.3274 vs WT. **D** Percentage (%) of alternations in Y-maze test. Unpaired t-test, *p* = 0.3460 vs WT. **E**–**H** Resident intruder test. **E** Lateral threat time, Unpaired t-test *p* = 0.7536 vs WT. **F** Upright posture time. Unpaired t-test, *p* = 0.3070 vs WT. **G** Attack latency. Unpaired t-test, *p* = 0.1284 vs WT. Data are presented as mean ± SEM. *n* = 10 (WT) and 7 (C1q KO).

To determine whether the C1q deletion induces any change in behavior, we performed several behavioral tests in C1q KO and WT mice. Social behavior was examined by three-chamber and reciprocal interaction tests. We did not find any significant difference in the time spent in the chamber between C1q KO and WT mice in the three-chamber test (Fig. 1A). Also, no significant difference was observed in the interaction time between C1q KO and WT mice in the reciprocal interaction test (Fig. 1B). In the tail suspension test, we found no significant difference in the immobility time between C1q KO and WT mice (Fig. 1C). Y-maze test was performed to assess the spatial memory function. The percentage of alternation was similar between C1q KO and WT mice in the Y-maze test indicating that C1q deletion does not affect spatial memory in mice (Fig. 1D). To examine if C1q deletion induces changes in aggressive behavior, we performed the resident intruder test. We scored the time duration of multiple aggressive behaviors including lateral threat, upright posture, and attack latency. No significant difference was found in aggressive behavior between C1q KO and WT mice (Fig. 1E–G). Together, these results suggest that C1q deletion does not result in any significant change in social interaction, despair behavior, spatial memory, and aggressive behavior in mice.

### C1q deletion exacerbates the stress-induced helplessness behavior in mice

**Fig. 2:**
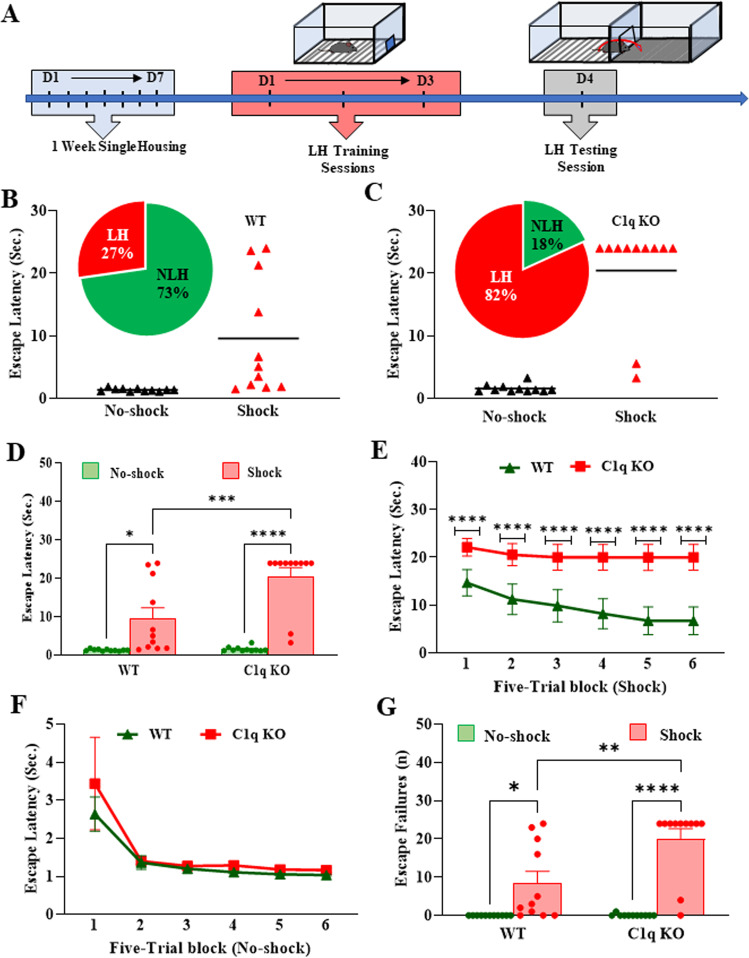
C1q deletion exacerbates the stress-induced helplessness behavior in mice. **A** Learned helplessness (LH) paradigm. **B**, **C** Percentage of mice exhibiting helplessness. **D** Mean escape latency in an escape test. Two-way ANOVA, genotype X shock interaction (F (1, 40) = 8.313, *p* = 0.0063); Tukey’s multiple comparisons; *p = 0.0161 vs no-shock WT, ***p = 0.0009 vs shock WT, ****p < 0.0001 vs no-shock C1q KO. Mean escape latency per five-trial block in **E** shock and **F** no-shock groups. **E** Two-way repeated-measures ANOVA, genotype X number of block interaction (F (5, 50) = 3.901, *p* = 0.0046); Tukey’s multiple comparisons; ****p < 0.0001 vs shock WT. **G** Number of escape failures. A trial was considered to be an escape failure when a mouse failed to escape a 0.3 mA foot shock within 24 s. Two-way ANOVA, genotype X shock interaction (F (1, 40) = 7.902, *p* = 0.0076); Tukey’s multiple comparisons; *p = 0.0236 vs no-shock WT, **p = 0.0014 vs shock WT, ****p < 0.0001 vs no-shock C1q KO. Data are presented as percentage (%) or mean ± SEM. *N* = 11 per group.

To determine whether the absence of C1q affects the LH behavior, we performed the LH paradigm using unpredictable EFSs (Fig. 2A). To evaluate the LH, mice were subjected to the shuttle-box escape task at 24 h after the last training session. During the training session, shock C1q KO mice showed significant increases in escape latency compared to the WT mice (Fig. 2B–F). The distribution analysis shows that there was a higher frequency of LH+ mice after the EFSs in the C1q KO mice group as compared to the WT mice (Fig. 2B, C). Further, the five-trial block analysis indicated that there was a persistent increase in the escape latency over each five-trial block throughout the 30 trials (Fig. 2E, F). In addition, shock C1q KO mice showed significant increases in the number of escape failures (Fig. 2G). These results indicate that the deletion of C1q promotes the learned helplessness behavior in mice.

### LH and NLH mice show reduced C1q expression in the PFC

**Fig. 3:**
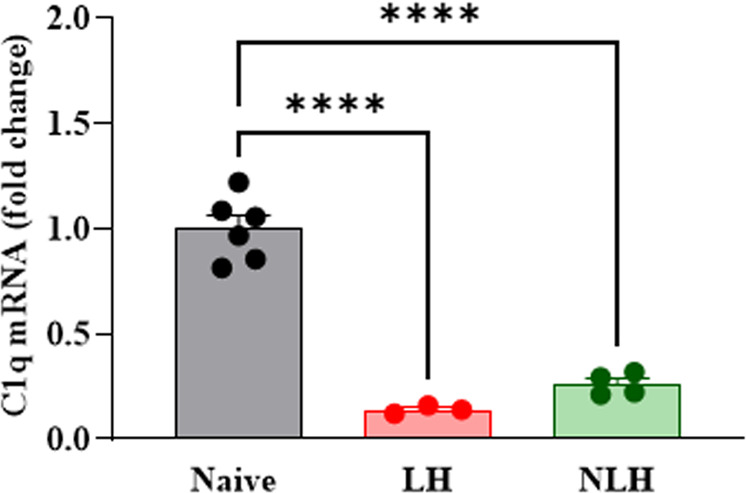
LH and NLH mice show reduced C1q expression in the PFC. C1q mRNA expression (fold change) in naïve (*n* = 6), LH (*n* = 3) and NLH (*n* = 4) WT mice. One-way ANOVA (F = 82.13, *p* < 0.0001); Tukey’s multiple comparisons; ****p < 0.0001vs naïve. Data are shown as mean ± SEM.

We examined whether LH is associated with changes in C1q expression in WT mice. We found that unpredictable EFSs resulted in a significant reduction in C1q mRNA in the PFC, a brain region highly implicated in mood and behavior. Interestingly, we also observed a reduction in C1q mRNA in the PFC of NLH mice as compared to naïve mice (Fig. 3).

### C1q deletion is associated with increased levels of pro-inflammatory cytokines in the PFC

**Fig. 4:**
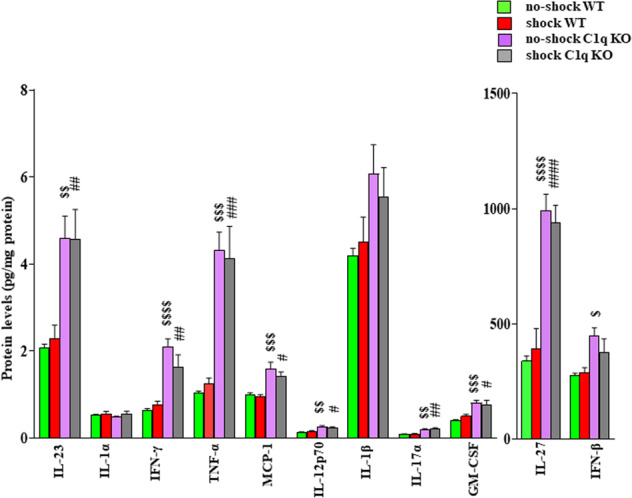
C1q deletion is associated with increased levels of pro-inflammatory cytokines in the PFC. IL-23. Two-way ANOVA, genotype X treatment interaction (F (1, 23) = 0.06640, *p* = 0.7989, *n* = 6–7); Tukey’s multiple comparisons; ^$$^*p* = 0.0056 vs no-shock WT, ^##^*p* = 0.0094 vs shock WT. IL-1α. Two-way ANOVA, genotype X treatment interaction (F (1, 23) = 0.2762, *p* = 0.6043, *n* = 6–7); Tukey’s multiple comparisons- no significant difference between the groups. IFN-γ. Two-way ANOVA, genotype X treatment interaction (F (1, 23) = 2.744, *p* = 0.1112, *n* = 6–7); Tukey’s multiple comparisons; ^$$$$^*p* < 0.0001 vs no-shock WT, ^##^*p* = 0.0081 shock WT. TNF-α. Two-way ANOVA, genotype X treatment interaction (F (1, 23) = 0.2117, *p* = 0.6497, *n* = 6–7); Tukey’s multiple comparisons; ^$$$^*p* = 0.0002 vs no-shock WT, ^###^*p* = 0.0005 shock WT. MCP-1. Two-way ANOVA, genotype X treatment interaction (F (1, 20) = 0.4140, *p* = 0.5273, *n* = 5–7); Tukey’s multiple comparisons; ^$$$^*p* = 0.0010 vs no-shock WT, ^#^*p* = 0.0165 vs shock WT. IL-12p70. Two-way ANOVA, genotype X treatment interaction (F (1, 23) = 1.207, *p* = 0.2833, *n* = 6–7); Tukey’s multiple comparisons; ^$$^*p* = 0.0013 vs no-shock WT, ^#^*p* = 0.0342 vs shock WT. IL-1β. Two-way ANOVA, genotype X treatment interaction (F (1, 23) = 0.5080, *p* = 0.4832, *n* = 6–7); Tukey’s multiple comparisons—no significant difference between the groups. IL-17α. Two-way ANOVA, genotype X treatment interaction (F (1, 23) = 0.0573, *p* = 0.8128, *n* = 6–7); Tukey’s multiple comparisons; ^$$^*p* = 0.0053 vs no-shock WT, ^##^*p* = 0.0016 vs shock WT. GM-CSF. Two-way ANOVA, genotype X treatment interaction (F (1, 23) = 1.087, *p* = 0.3079, *n* = 6–7); Tukey’s multiple comparisons; ^$$$^*p* = 0.0009 vs no-shock WT, ^#^*p* = 0.0206 vs shock WT. IL-27. Two-way ANOVA, genotype X treatment interaction (F (1, 24) = 0.5738, *p* = 0.4561, *n* = 7); Tukey’s multiple comparisons; ^$$$$^*p* < 0.0001 vs no-shock WT, ^####^*p* < 0.0001 vs shock WT. INF-β. Two-way ANOVA, genotype X treatment interaction (F (1, 24) = 1.407, *p* = 0.2472, *n* = 6–7); Tukey’s multiple comparisons; ^$^*p* = 0.0114 vs no-shock WT. Data are shown as mean ± SEM.

Changes in cytokine levels have been well-implicated in the pathophysiology of depression [32]. In addition, C1q has been shown to regulate inflammatory cytokines and inflammation [33]. Based on these findings, we examined the effects of C1q deletion and unpredictable EFSs on cytokine levels in the PFC and serum. We performed the cytokine array of 13 cytokines in the PFC and serum samples of shock and no-shock WT and C1q KO mice. Out of 13 cytokines, IL-10 and IL-6 were not detected in the PFC samples whereas MCP-1, IL-17α, GM-CSF, and IL-10 were not detected in the serum samples. We found that the levels of IL-23, IFN-γ, TNF-α, MCP-1, IL-12p70, IL-17α, GM-CSF, and IL-27 were significantly increased in the PFC of no-shock as well as shock C1q KO mice as compared to the corresponding WT mice (Fig. 4). Also, we found a significant increase in IFN-β levels in no-shock C1q KO mice as compared to no-shock WT mice (Fig. 4). No significant difference in the levels of the above cytokines was observed between no-shock and shock groups. In serum samples, TNF-α was significantly increased in the shock C1q KO mice as compared to the shock WT mice (Fig. S1). No significant change in other serum cytokine levels was found between groups.

### Shock treatment results in increases in macrophage/microglia M1 markers in the PFC of C1q KO mice

**Fig. 5:**
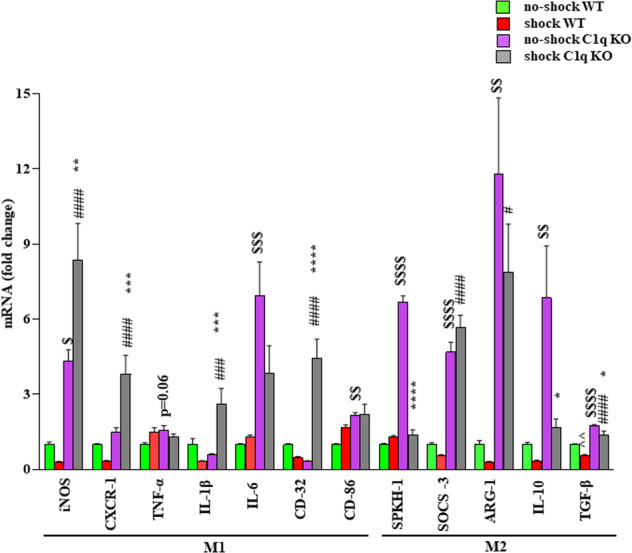
Shock treatment results in increases in macrophage/microglia M1 markers in the PFC of C1q KO mice. *mRNA expression (fold change) of the M1 markers*: iNOS. Two-way ANOVA, genotype X treatment interaction (F (1, 22) = 11.34, *p* = 0.0028, *n* = 6–7); Tukey’s multiple comparisons; ^$^*p* = 0.0143 vs no-shock WT, ^####^*p* < 0.0001 vs shock WT, **p = 0.0039 vs no-shock C1q KO. CxCR-1. Two-way ANOVA, genotype X treatment interaction (F (1, 22) = 17.22, *p* = 0.0004, *n* = 6–7); Tukey’s multiple comparisons; ^####^*p* < 0.0001 vs shock WT, ***p = 0.0008 vs no-shock C1q KO. TNF-α. Two-way ANOVA, genotype X treatment interaction (F (1, 23) = 5.830, *p* = 0.0241, *n* = 6–7); Tukey’s multiple comparisons; *p* = 0.06 vs shock WT. IL-1β. Two-way ANOVA, genotype X treatment interaction (F (1, 22) = 17.81, *p* = 0.0004, *n* = 6–7); Tukey’s multiple comparisons; ^###^*p* = 0.0004 vs shock WT^, *^*p = 0.0001 vs no-shock C1q KO. IL-6. Two-way ANOVA, genotype X treatment interaction (F (1, 22) = 3.821, *p* = 0.0634, *n* = 6–7); Tukey’s multiple comparisons; ^$$$^*p* = 0.0005 vs no-shock WT. CD-32. Two-way ANOVA, genotype X treatment interaction (F (1, 23) = 47.52, *p* < 0.0001, *n* = 6–7); Tukey’s multiple comparisons; ^####^*p* < 0.0001 shock WT, ****p < 0.0001 vs no-shock C1q KO. CD-86. Two-way ANOVA, genotype X treatment interaction (F (1, 21) = 2.554, *p* = 0.2054, *n* = 6–7); Tukey’s multiple comparisons; ^$$^*p* = 0.0027 vs no-shock WT. *mRNA expression (fold change)* *of the M2 markers*: SPKH-1. Two-way ANOVA, genotype X treatment interaction (F (1, 22) = 299.5, *P* < 0.0001, *n* = 6–7); Tukey’s multiple comparisons; ^$$$$^*p* < 0.0001vs no-shock WT, ****p < 0.0001 vs no-shock C1q KO. SOCS-3. Two-way ANOVA, genotype X treatment interaction (F (1, 23) = 6.089, *p* = 0.0215, *n* = 6–7); Tukey’s multiple comparisons; ^$$$$^*p* < 0.0001 vs no-shock WT, ^####^*p* < 0.0001 vs shock WT. ARG-1. Two-way ANOVA, genotype X treatment interaction (F (1, 23) = 0.7882, *p* = 0.3838, *n* = 6–7); Tukey’s multiple comparisons; ^$$^*p* = 0.0014 vs no-shock WT, ^#^*p* = 0.0317 vs shock WT. IL-10. Two-way ANOVA, genotype X treatment interaction (F (1, 22) = 3.834, *p* = 0.0630, *n* = 5–7); Tukey’s multiple comparisons; ^$$^*p* = 0.0054 vs no-shock WT, *p = 0.0281 vs no-shock C1q KO. TGF-β. Two-way ANOVA, genotype X treatment interaction (F (1, 22) = 0.2904, *p* = 5954, *n* = 6–7); Tukey’s multiple comparisons; ^^*p* = 0.0036 vs no-shock WT, ^$$$$^*p* < 0.0001 vs no-shock WT, ^####^*p* < 0.0001 vs shock WT, *p = 0.021 vs no-shock C1q KO.

Previous studies have shown that the microglia are the major source of C1q in the brain [34]. Further, microglia switch between pro-(M1) and anti-inflammatory (M2) phenotypes as a result of disturbed brain homeostasis [35–37]. We analyzed the mRNA levels of M1 markers, Inducible nitric oxide synthase (iNOS), C-X3-C Motif Chemokine Receptor-1 (Cx3CR1), TNFα, IL-1β, IL-6, CD-32 and CD-86, and M2 markers, Sphingosine kinase-1 (Spkh-1), Suppressor of cytokine signaling-3 (Socs-3), and Arginase-1 (Arg-1), IL-10 and transforming growth factor-beta (TGF-β) in the PFC of WT and C1q KO mice following shock or no-shock treatment. The q-PCR analysis shows that C1q deletion increased the mRNA levels of iNOS, IL-6, and CD-86 (M1 markers), and Spkh-1, Socs-3, Arg-1, IL-10, and TGF-β (M2 markers) in the PFC of no-shock mice (Fig. 5). Similarly, the mRNA levels of iNOS, CXCR-1, IL-1β, and CD-32 (M1 markers), and SOCS-3, and TGF-β (M2 markers) were significantly increased in the PFC of shock C1q KO mice as compared to shock WT mice (Fig. 5). Also, shock treatment induced significant decreases in the mRNA levels of TGF-β (M2 marker) in the PFC of WT mice. We found significant increases in M1 markers such as iNOS, CXCR-1, IL-1β, and CD-32 in the PFC of shock C1q KO mice as compared to no-shock C1q KO mice. However, the mRNA levels of M2 markers such as Spkh-1, IL-10, and TGF-β were significantly lower in the PFC of shock C1q KO mice as compared to no-shock C1q KO mice (Fig. 5).

## Discussion

The LH model is one of the widely used models to study stress-induced depressive-like behavior in rodents [38, 39]. Our findings showed that LH mice have decreased mRNA expression of C1q in the PFC as compared to the naïve WT mice. C1q deletion exacerbates the inescapable EFS-induced LH behavior in mice. Further, C1q deletion is associated with increased levels of pro-inflammatory markers in the PFC.

The innate immune system is the first line of host defense system which reacts primarily against the external pathogens [40]. The defense mechanism of innate immune system is mediated by several immune cells, molecules as well as the complement system. The complement system is regulated by the activation of one of the three distinct pathways; classical pathway, alternative pathway, and lectin pathway. Besides the various functions of the classical complement pathway, C1q is also involved in facilitating the chemotaxis [41], cellular differentiation [42], intercellular adhesion [43], aggregation of cellular macromolecules [43], and clearance of apoptotic cell debris [44]. Further, C1q has been shown to bind β-amyloid fibrils resulting in the activation of the complement pathway [45].

In the present study, we observed no significant difference in social behavior, behavioral despair, spatial memory, and aggressive behavior between WT and C1q KO mice suggesting that C1q deletion does not affect these behavioral measures in mice. However, C1q deletion exacerbated the EFS-induced LH behavior in mice. LH is an induced depressive-like behavior. The difference between LH and other behavior tests performed in this study is that the animals were not exposed to a stressor prior to testing in the other behavior tests. Such a strategy provided the baseline data related to social interaction, spatial memory, and aggressive behavior in C1q KO mice. It needs to be noted that we have not separated the shock mice into LH and NLH groups because it is known that both LH and NLH mice exhibit increased anhedonia, anxiety, and uncontrollable EFS-induced depressive-like behavior, irrespective of the state of helplessness [46]. Moreover, we were not able to perform a comparative analysis between the LH and NLH mice since we did not find a sufficient number (*n*) of LH and NLH animals in WT and C1q KO groups, respectively.

Increasing evidence suggest the role of inflammatory pathways in depression. Studies show that pro-inflammatory cytokines can impair hippocampal-dependent memory [47, 48] and promote depressive-like behaviors in mice [32, 49]. In addition, psychological stress conditions are known to induce pro-inflammatory cytokines [32, 50]. Although many complement proteins are known to promote inflammatory pathways, evidence also indicates that they can inhibit the production of pro-inflammatory cytokines in a cell-dependent manner [51, 52]. In this regard, C1q independent from the C1 complex has been shown to downregulate the in vitro production of pro-inflammatory cytokines [53–55]. Also, C1q suppressed lipopolysaccharide-induced pro-inflammatory cytokines in human monocytes [33]. C1q stimulation has been shown to activate p50/p50 homodimers resulting in reduced NFκB-mediated transcription of pro-inflammatory cytokines in monocytes [56]. In addition, increased CREB phosphorylation and thereby a competition with p65/p50 for limited amounts of CBP has also been implicated as a mechanism underlying C1q-induced reduction in pro-inflammatory cytokine transcription [56]. In agreement, we found significant increases in the levels of a number of pro-inflammatory cytokines in the PFC of C1q KO mice. However, future studies should investigate the mechanisms underlying C1q-mediated regulation of inflammatory cytokines following stress conditions.

A number of studies have reported altered cytokine levels in the brain of depressed subjects. Increased protein levels of trans-membrane TNF were found in the Brodmann area 46 (BA46), but not in BA24 in the PFC of MDD subjects [57]. However, no significant change in soluble TNF was found in BA24 or BA46 between the MDD and control subjects [57]. Significant increases in the protein and mRNA levels of IL-1β and IL-receptors were reported in postmortem frontal cortex from BD patients compared with control subjects [58]. Pandey et al. (2012) found increases in protein and mRNA expression of IL-1β, IL-6, and TNF-α in the PFC (BA10) of teenage suicide victims compared with normal control subjects [59]. Further, mRNA and protein expression levels of TNF-α, IL-1β, and IL-6 were significantly higher in the PFC of MDD and BD subjects [60]. Microarray analysis showed up-regulation of a number of pro- and anti-inflammatory cytokines, including IL-1α, IL-2, IL-3, IL-5, IL-8, IL-9, IL-10, IL-12A, IL-13, IL-15, IL-18, IFNγ, and lymphotoxin α (TNF superfamily member 1) in the BA10 of psychotropic drug-free persons with a history of MDD [61]. In contrast, Tonelli et al. (2008) did not find a significant difference in the mRNA expression of IL-1β, IL-6, and TNF-α in postmortem frontal cortex (BA11) between suicide and normal controls [62]. Together, the above postmortem brain studies indicate that the levels of inflammatory cytokines vary depending on a number of factors including the brain region, sample characteristics and/or the methodology used in the analysis. Although a number of previous studies have reported increases in the levels of pro-inflammatory markers in the brain tissues of animals following stress conditions [63], we did not find any significant change in the levels of pro-inflammatory cytokines in the PFC of WT mice following shock treatment. These differences may be related to the rodent model of depression used and/or the brain region analyzed.

Microglia are the primary resident myeloid cells of the mammalian central nervous system (CNS) parenchyma and mediate normal brain development and homeostasis. Microglial phenotypes are divided into M1 with pro-inflammatory activity [64] and M2 with anti-inflammatory activity [65]. Like pro-inflammatory cytokine levels in the PFC, the levels of M1 markers such as iNOS, IL-6, and CD-86 were found increased in the PFC of C1q KO mice. However, no significant change in M1 markers was found in the PFC of WT mice following shock treatment. Interestingly, shock treatment resulted in significant increases in the levels of M1 markers in C1q KO mice suggesting that C1q KO mice are more sensitive to shock treatment-induced pro-inflammatory M1 phenotype. Further, our findings on the increased levels of M1 markers are consistent with previous findings suggesting a significant association between M1 phenotype of microglia and depressive-like behaviors [66]. C1q deletion also induced increases in M2 markers in the PFC as compared to WT mice. Although an M2 response is required to downregulate the inflammation, M2 microglia do not exert beneficial effects in all conditions [67–70]. The complex nature of M2 microglia suggests that their response depends on the cells in the context and the nature of the insult [70]. Therefore, additional studies are warranted to differentiate the M1/M2 dynamics between resident macrophages (microglia) and infiltrating macrophages in our model. In this regard, we found increased levels of MCP-1 in the PFC of C1q KO mice. MCP-1 is known to compromise the integrity of BBB suggesting the possibility of monocyte/macrophage recruitment into the PFC of C1q KO mice. A significant increase in MCP-1 mRNA has been reported in the dorsal anterior cingulate cortex of depressed suicide subjects compared with normal control subjects [71]. However, two other studies reported a decrease in the gene expression of MCP-1 in BA9 in the PFC of depressed suicide subjects [72, 73]. Since chemokines such as MCP-1 are also involved in a number of CNS functions including neurogenesis [74] and angiogenesis [75] which are implicated in depression, their roles in functions beyond chemotaxis need further investigation.

Although our findings provide the first evidence on the role of C1q in stress-induced depressive-like behavior, a few limitations of this study need to be noted. The cytokine and M1/M2 analyses were performed in only PFC samples. Additional brain regions including hippocampus and amygdala should be included in future studies. Also, the effects of shock on behaviors other than LH in C1q KO mice need to be investigated. Our study used global C1q KO mice which do not address the cell specific role of C1q in mediating depressive-like behavior. Our previous study has shown that inhibition of C3 signaling attenuates chronic unpredictable stress-induced depressive-like behavior in mice [24]. Further, we found that inhibition of C3aR1 blocks CUS-induced infiltration of macrophages into the PFC [24]. In contrast, our current findings show that C1q deletion promotes the levels of proinflammatory cytokines in the PFC. It is important to note that the stress paradigms used in these two studies are different. Therefore, additional studies investigating the role of C3 in the LH model are necessary to further understand whether C1q and C3 act differently in mediating stress-induced depressive-like behavior.

## Supplementary information

Supplementary information

Table S1

Figure S1
