# Supplementary material for: C1q deletion exacerbates stress-induced learned helplessness behavior and induces neuroinflammation in mice
Source: Transl Psychiatry. 2022 Feb 1;12:50. doi: 10.1038/s41398-022-01794-4 (PMC8807734; doi:10.1038/s41398-022-01794-4)
Supplement: Supplementary file 1 — Supplementary information [file 41398_2022_1794_MOESM1_ESM.docx]

**Supplementary information**

**Figure S1. Increase in serum TNF-α levels in shock C1q KO mice.** TNF-α. Two-way ANOVA, genotype X treatment interaction (F (1, 19) = 17.66, p=0.0005, n=5-6); Tukey’s multiple comparisons; ^##^p=0.0091 vs shock WT.

**Table S1: Mouse Primer sequences**
