# Supplementary material for: C1q deletion exacerbates stress-induced learned helplessness behavior and induces neuroinflammation in mice
Source: Transl Psychiatry. 2022 Feb 1;12:50. doi: 10.1038/s41398-022-01794-4 (PMC8807734; doi:10.1038/s41398-022-01794-4)
Supplement: Supplementary file 2 — Table S1 [file 41398_2022_1794_MOESM2_ESM.docx]

**Table S1: Mouse Primer sequences**

| **Gene** | **Forward Primer** | **Reverse Primer** |
| --- | --- | --- |
| C1q | ATG GAG ACC TCT CAG GGA TG | ATA CCA GTC CGG ATG CCA GC |
| iNOS | CCC TTC AAT GGT TGG TAC ATG G | ACA TTG ATC TCC GTG ACA GCC |
| Cxc3cr1 | AAG TTC CCT TCC CAT CTG CT | GGA CAG GAA GAT GGT TCC AA |
| TNF-α | CGT CAG CCG ATT TGC TAT CT | CGG ACT CCG CAA AGT CTA AG |
| IL-1β | TGT AAT GAA AGA CGG CAC ACC | TCT TCT TTG GGT ATT GCT TGG |
| IL-6 | GCT ACC AAA CTG GAT ATA ATC AGG A | CCA GGT AGC TAT GGT ACT CCA GAA |
| CD-32 | CTG GAA GAA GCT GCC AAA AC | CCA ATG CCA AGG GAG ACT AA |
| CD-86 | GAG CGG GAT AGT AAC GCT GA | GGC TCT CAC TGC CTT CAC TC |
| SPKH-1 | TCC AGA AAC CCC TGT GTA GC | CAG CAG TGT GCA GTT GAT GA |
| SOCS-3 | CGT TGA CAG TCT TCC GAC AA | TAT TCT GGG GGC GAG AAG AT |
| ARG-1 | GTG AAG AAC CCA CGG TCT GT | GCC AGA GAT GCT TCC AAC TG |
| IL-10 | GGC TGA GGC GCT GCT ATC G | TCA TTC ATG GCC TTG TAG ACA CC |
| TGF-β | CTT TTG ACG TCA CTG GAG TTG | CAG TGA GCG CTG AAT CGA A |
| B2M | CCC CAC TGA GAC TGA TAC ATA CG | CGA TCC CAG TAG ACG GTC TTG |
