# Supplementary figures and images for: C1q deletion exacerbates stress-induced learned helplessness behavior and induces neuroinflammation in mice
Source: Transl Psychiatry. 2022 Feb 1;12:50. doi: 10.1038/s41398-022-01794-4 (PMC8807734; doi:10.1038/s41398-022-01794-4)

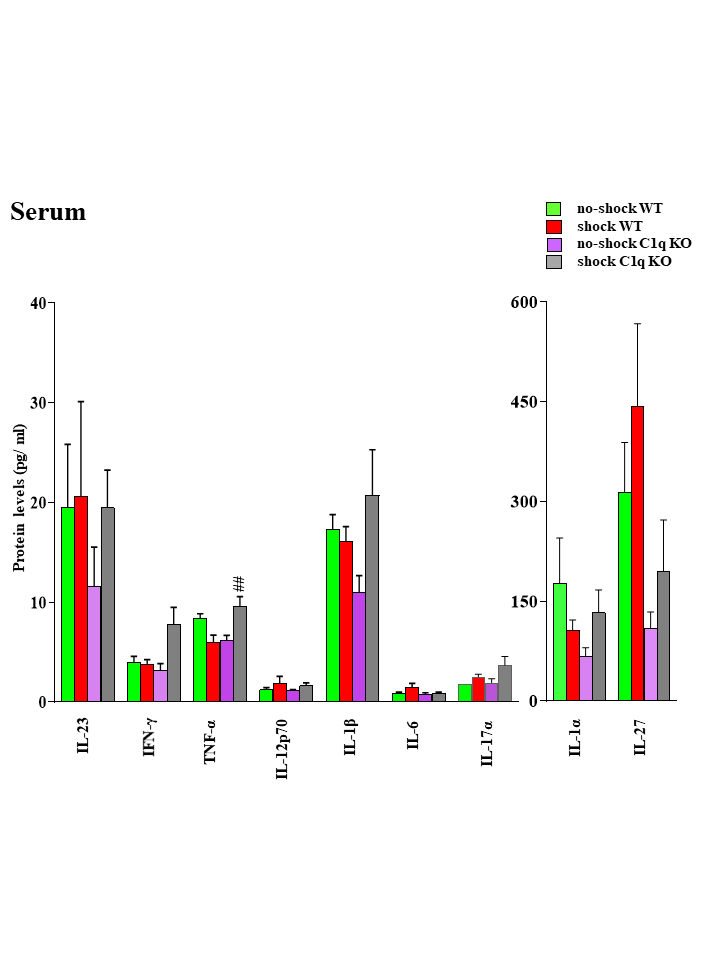

Supplement: Supplementary file 3 — Figure S1 [file 41398_2022_1794_MOESM3_ESM.tif]
